# Inhibition of *Listeria monocytogenes* on Ready-to-Eat Meats Using Bacteriocin Mixtures Based on Mode-of-Action

**DOI:** 10.3390/foods6030022

**Published:** 2017-03-14

**Authors:** Paul Priyesh Vijayakumar, Peter M. Muriana

**Affiliations:** 1 Department of Animal and Food Science, University of Kentucky, 213 W.P. Garrigus Building, Lexington, KY 40546-0215, USA; paul.v@uky.edu; 2 Robert M. Kerr Food & Agricultural Products Centre, Oklahoma State University, 109 FAPC Building, Monroe Street, Stillwater, OK 74078-6055, USA

**Keywords:** *Listeria monocytogenes*, ready-to-eat meats, bacteriocin, mode-of-action, biopreservatives

## Abstract

Bacteriocin-producing (Bac^+^) lactic acid bacteria (LAB) comprising selected strains of *Lactobacillus curvatus*, *Lactococcus lactis*, *Pediococcus acidilactici*, and *Enterococcus faecium* and *thailandicus* were examined for inhibition of *Listeria monocytogenes* during hotdog challenge studies. The Bac^+^ strains, or their cell-free supernatants (CFS), were grouped according to mode-of-action (MOA) as determined from prior studies. Making a mixture of as many MOAs as possible is a practical way to obtain a potent natural antimicrobial mixture to address *L. monocytogenes* contamination of RTE meat products (i.e., hotdogs). The heat resistance of the bacteriocins allowed the use of pasteurization to eliminate residual producer cells for use as post-process surface application or their inclusion into hotdog meat emulsion during cooking. The use of Bac^+^ LAB comprising 3× MOAs directly as co-inoculants on hotdogs was not effective at inhibiting *L. monocytogenes.* However, the use of multiple MOA Bac^+^ CFS mixtures in a variety of trials demonstrated the effectiveness of this approach by showing a >2-log decrease of *L. monocytogenes* in treatment samples and 6–7 log difference vs. controls. These data suggest that surface application of multiple mode-of-action bacteriocin mixtures can provide for an Alternative 2, and possibly Alternative 1, process category as specified by USDA-FSIS for control of *L. monocytogenes* on RTE meat products.

## 1. Introduction

*Listeria monocytogenes* is a formidable foodborne pathogen that causes listeriosis which results in high hospitalization rates (>90%) and mortalities (20%–30%) in large outbreaks [1]. Vulnerable populations include immuno-compromised, the sick and elderly, pregnant women, and infants. *Listeria monocytogenes* is associated with numerous animals [2] and therefore may be found as a ubiquitous contaminant on many animal-derived raw food products and ingredients that helps the organism find its way into meat and poultry processing facilities. The United States Department of Agriculture’s Food Safety and Inspection Service (USDA-FSIS) found incidences as high as 7.24% on small cooked sausages (i.e., hotdogs; 1991) and 7.69% on sliced ham and luncheon meats (1996) in nationwide sampling program for ready-to-eat meats (RTE) in the early 1990’s [3]. Their ability to remain as a persistent problem in RTE meat processing plants is a combination of their steady influx on raw ingredients as well as their ability to form biofilms that may resist sanitation efforts and allow the organism to be a persistent contaminant [4,5].

The RTE meat industry has been constantly battling the occurrence of *L. monocytogenes*. While RTE meats primarily rely on salt, curing agents, and refrigerated storage for microbial stability and safety, *Listeria* can capitalize on these conditions by growth at low temperatures and high salt concentrations. The CDC reported that *L. monocytogenes* is responsible for 2500 illness cases and 500 deaths annually (www.cdc.gov/ncidod/disease/foodborn/lister.htm). Hotdogs have maintained a designation as a high-risk RTE meat for *L. monocytogenes* because of high contamination rates [6]. Contamination occurs on the surface of the product during post-process exposure and steps such as peeling and packaging are potential routes for pathogen entry. The primary hurdle against foodborne pathogens and bacterial contamination in the food industry includes preventive measures such as good manufacturing practices (GMPs) and standard operating procedures (SOPs) in addition to a hazard analysis and critical control point (HACCP) food safety plan required for meat and poultry products [7,8]. Though the food industry incorporates a variety of precautionary measures, outbreaks due to foodborne illness continue to occur periodically. Therefore, there is a need for effective antimicrobials that may continue to provide food safety protection during shelf life and distribution of sensitive products.

The lactic acid bacteria (LAB) are well known for producing antimicrobials including organic acids, diacetyl, acetoin, hydrogen peroxide, reuterin, reutericyclin, antifungal peptides, and bacteriocins [9,10,11]. Although lactic acid is one of the most common acidulants, there has been considerable interest and research in the field of bacteriocins with respect to use of bacteriocinogenic (Bac**^+^**) LAB cultures or bacteriocin-containing culture fermentates as food preservatives [12]. Bacteriocin-producing cultures have been proposed as protective cultures to combat foodborne pathogens and spoilage bacteria in food systems [13,14,15,16,17]. The addition of bacteriocins includes the use of partially purified Bac^+^ preparations [18] or pre-cultured bacteriocin-containing (Bac^+^) cell-free supernatants (CFS) obtained from Bac^+^ LAB [19] as food ingredients. While the addition of purified bacteriocins as food preservatives needs regulatory approval and must be treated as direct food additives, the inclusion of Bac^+^ CFS from LAB cultures do not have the same regulatory restrictions [12].

In this study, we examined the effectiveness of Bac^+^ LAB and Bac^+^ CFS mixtures to prevent the growth of *L. monocytogenes* on RTE meats (hotdogs). Our approach included mixtures of bacteriocins demonstrating different modes-of-action (MOA), or the strains that produce them, that could provide enhanced efficacy against *L. monocytogenes* as opposed to preparations having a single MOA that could allow the development of spontaneous bacteriocin-resistant *L. monocytogenes* [20,21,22].

## 2. Materials and Methods

### 2.1. Bacterial Cultures

Strains of LAB were cultured at 30 °C in Lactobacilli MRS broth (Difco™, Becton-Dickenson Laboratories, Sparks, MD, USA) while *L. monocytogenes* 39-2, an isolate from retail hotdogs [23], was cultured in tryptic soy broth (TSB, Difco™) at 30 °C. Enumeration of LAB from either Bac^+^ LAB-*L. monocytogenes* hotdog challenge studies, or as LAB contaminants in Bac^+^ CFS-*Listeria* challenge studies, was done using MRS agar adjusted with HCl to pH 5.4–5.5 prior to autoclaving (the pH was found to be ~pH 5.5–5.7 after autoclaving) [24]. Acidified MRS agar inhibited growth of *L. monocytogenes* 39-2 but allowed the growth of LAB as determined from prior studies. *Listeria monocytogenes* 39-2 was selectively enumerated on MOX agar (Modified Oxford agar, Difco™) which was inhibitory to LAB. The *L. monocytogenes* 39-2 strain used in this study is resistant to 50 µg/mL of both streptomycin and rifamycin (Mediatech, Inc., Herndon, VA, USA); plate counts of *L. monocytogenes* 39-2 were occasionally confirmed on TS agar containing antibiotics. Bacterial cultures used in this study are listed in Table 1. Several Bac^+^ cultures (FS56-1, FS92) were previously identified as *Lactococcus lactis* and grew well and made bacteriocins in MRS media; however, during the course of our studies they were identified by 16S rRNA PCR/sequencing to be *Enterococcus* sp. (Table 1).

### 2.2. Bacteriocin Preparations

Bacteriocins were prepared by 2× repetitive transfer of individual Bac^+^ LAB overnight at 30 °C followed by centrifugation at 20,000× *g* (rcf) for 10 min at 4 °C (Sorvall RC50 Plus, ThermoFisher Scientific, ‎Waltham, MA, USA). The supernatants were carefully decanted to sterile bottles and filter-sterilized through 0.22 μ cellulose acetate syringe filters (VWR, Radnor, PA, USA) or pasteurized at 80 °C for 15 min. Bacteriocin preparations were then stored at 4 °C, or frozen at −20 °C if not expected to be used within a few days. Each of the filter-sterilized or pasteurized Bac^+^ CFS preparations were also plated on MRSA plates, or into MRS broth, and incubated (30 °C) in order to check the effectiveness of the pasteurization or filter-sterilization process (i.e., no growth).

### 2.3. Manufacture of Hotdogs for Bacteriocin Applications

**Figure 1:**
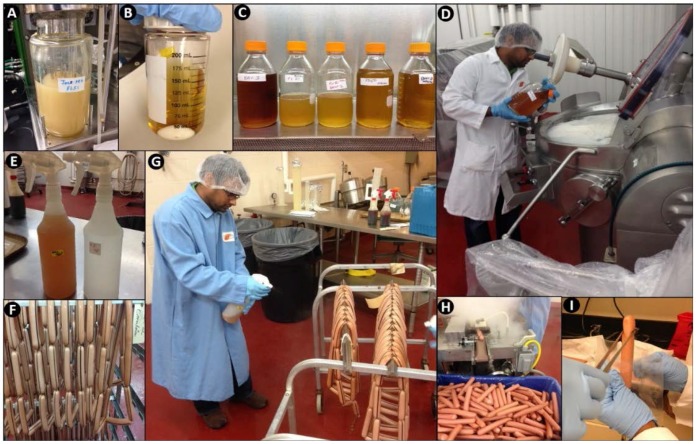
Manufacture of hotdogs for bacteriocin treatment. (**A**) Culturing microorganisms; (**B**) centrifugation of Bac^+^ supernatants; (**C**) pasteurized Bac^+^ CFS; (**D**) addition of Bac^+^ CFS mixture to hotdog meat matrix (Trial #2); (**E**) spray bottles with pasteurized Bac^+^ CFS; (**F**) pre-cooked hotdogs in casings; (**G**) spraying cooked hotdogs in casings with Bac^+^ CFS mixture (Trial #2); (**H**) hotdogs after peeling; (**I**) addition of hotdogs to vacuum package bags for addition of Bac^+^ CFS and the *L. monocytogenes* inoculum (Trials #3, #4, and #5).

Hotdogs were manufactured in-house for use in shelf life trials (Figure 1). Beef and pork trimmings were used to manufacture hotdogs in the Meat Pilot Plant in the R.M. Kerr Food and Ag Products Center (FAPC) at Oklahoma State University, Stillwater, OK. Hotdogs were manufactured with the following formulation (per 35.52 lbs): beef (81% lean; 4.5 lbs), pork (72% lean; 13.25 lbs), pork (42% lean; 7.25 lbs), water/ice (6.25%; 9.45 lbs), Legg’s Bolo seasoning (1.0 lb), cure (6.25% nitrite; 0.06 lb), and sodium erythorbate (0.01 lbs). Antimicrobials such as lactate and diacetate were not added, as is commonly done in commercial frankfurters, so as not to confuse the source of antimicrobial activity during bacteriocin treatments. Emulsions were stuffed into Viscofan 24/USA casings and thermally processed (cooked) in an electric-fired, batch oven (Alkar, DEC International, Washington, DC, USA) to an internal temperature of 88 °C (190 °F). After cooking, hotdogs in casings were chilled with a cold water rinse and then peeled using a peeling machine (PS760L Peeler, Linker Machines, Rockaway, NJ, USA). The formulation above was used for surface-treatment with Bac^+^ CFS or Bac^+^ LAB applied prior to packaging. Additional hotdog formulation modifications included replacement of the added water with pasteurized Bac^+^ CFS, the use of chilled pasteurized Bac^+^ CFS spray warm hotdogs still in casings, and surface application of CFS during packaging (Figure 1). The hotdogs manufactured by these different protocols were kept separate from each other, vacuum packaged, and stored frozen until used.

### 2.4. Hotdog Challenge Studies

#### 2.4.1. Preliminary Treatment of Hotdogs Prior to Challenge Studies

Hotdogs manufactured for use in challenge studies were stored frozen in a blast chiller (−26 °C) in single-layer packages. They were then thawed prior to use and pasteurized by dipping packages into a temperature-controlled, steam-injected 50-gal hot water bath at 82 °C for 5 min in order to eliminate any indigenous bacterial contaminants that could have been acquired during post-process handling. Hotdogs were then aseptically removed from the vacuum packages for use in experimental treatments.

#### 2.4.2. Trial #1: Application of Mixed Mode-of-Action (MOA) Bac^+^ LAB Co-Inoculated with *L. monocytogenes* in Shelf Life Challenge Studies

Selected Bac^+^ LAB cultures covering 3 different MOA were used in these trials: *Pe. acidilactici* Bac3 (pediocin Bac3), *En. faecium* FS56-1 (enterocin FS56), and *En. thailandicus* FS92 and RP-1 (enterocins FS92 and RP-1). Freshly grown overnight Bac^+^ LAB cultures were prepared to approximately similar levels by dilution in sterile 0.1% BPW and mixed in equal proportions prior to use. Thawed hotdogs were pasteurized as described above, and then immersed in the Bac^+^ culture mixture for 30 s using a sterile plastic basket and allowed to drain for 30 s before hotdogs were removed with sterile tongs to sterile vacuum packaging bags. A level of mixed Bac^+^ culture was used for dipping that would achieve approximately ~10^5^ cfu/mL in a recovered minimal hotdog rinse as determined by prior enumeration studies. Similarly, *L. monocytogenes* 39-2 was prepared by dilution in sterile 0.1% BPW and 100 μL was inoculated directly into the bagged hotdogs at a level resulting in recovery of *L. monocytogenes* at approximately ~10^4^ cfu/mL from the same minimal rinse recovery solutions. The hotdogs in the vacuum bags were then massaged to distribute *L. monocytogenes* and then vacuum-packaged. Bags were stored at 5 °C and sampled at 0 h, 3 days, and weekly at 1, 2, 3, 4, 5, 6 and 7 weeks. Triplicate replications of each treatment (3 bags/treatment) were sampled at the time intervals mentioned above. During sampling, each package was opened by snipping open the top corner, and a pipette was used to deliver 3 mL of diluent (0.1% BPW). The bags were hand-massaged and then a pipette was used to withdraw the contents into a sterile disposable plastic tube that was kept on ice; this was considered the 10° dilution. Further dilutions were made with 0.1% BPW and plated on acidified MRS agar (for LAB) or MOX agar (*L. monocytogenes*). A series of negative control samples containing only *L. monocytogenes* 39-2 were also included.

#### 2.4.3. Trial #2: Application of Mixed MOA Bacteriocin Preparations Added during the Manufacture of Hotdogs

Hotdogs were manufactured in the FAPC Meat Pilot Plant. Four 25-lb batches of hotdogs were manufactured which would be used in a variety of trials involving different formulations or treatments. Bacteriocin CFS preparations were obtained as described previously (cultured, centrifuged to remove cells, and pasteurized) and mixed in equal volumes: curvaticin FS47, curvaticin Beef3, lacticin FLS1, and pediocin Bac3 representing 3 different MOAs.

Control batches of hotdogs did not receive a bacteriocin application; these were also used in subsequent trials for surface application of a mixed bacteriocin cocktail prior to packaging (see next section). In the current trial, the mixed bacteriocin CFS preparation was added in place of the water component (9.45 lbs) in the raw meat emulsion. In an additional formulation and treatment within this trial, some hotdogs were sprayed after the cook process while still in casings instead of adding the CFS to the meat matrix. Sprayed Bac^+^ CFS preparation was allowed to absorb onto the permeable casing for up to 30 min after which the hotdog casings were peeled and the hotdogs were vacuum packaged and stored in a blast freezer at −26 °C. Prior to use in experiments, hotdogs were thawed and then pasteurized as described earlier. Hotdogs from these various treatments were then processed identically: they were placed in vacuum packaging bags (2 hotdogs/bag) with sterile tongs, inoculated with 100 μL of *L. monocytogenes* 39-2, hand massaged to evenly distribute the inoculum, and vacuum packaged. The bags were stored at 5 °C and sampled at 0 h, 3 days and weekly at 1, 2, 4, 6, 8, 10, and 12 weeks. Samples were plated on acidified MRS agar (pH 5.5) for enumerating LAB (if present) or MOX agar for enumerating *L. monocytogenes* 39-2.

#### 2.4.4. Trial #3: Application of Mixed Mode-of-Action Bac^+^ CFS on the Surface of RTE Meats (Hotdogs)

Select Bac^+^ CFS mixtures comprising 3 MOAs were obtained from *Lb. curvatus* FS47, *Lb. curvatus* Beef3, *Pe. acidilactici* Bac3, *En. faecium* FS56-1, *En. thailandicus* FS92, and/or *Lc. lactis* FLS1. Cultures were propagated, centrifuged, and CFS was processed as described earlier. Equal volumes of each CFS was mixed in a sterile tube to obtain a Bac^+^ CFS mixture comprising 3 MOA; different bacteriocins were used to obtain the 3 MOA mixture as indicated in the analogous figure legends. As before, hotdogs were pasteurized as described earlier. Using sterile tongs, pasteurized hotdogs were placed in vacuum packaging bags (2 hotdogs/bag) to which 300-μL of sterile water (control) or Bac^+^ CFS was added, massaged, and then inoculated with 100-μL of *L. monocytogenes* 39-2, hand-massaged again to distribute the inoculum, and then vacuum sealed. Samples were then stored at 5 °C and sampled at 0 h, 3 days and weekly at 1, 2, 4, 6, 8, 10, and 12 weeks. Samples were plated on acidified MRS agar (LAB) or MOX agar (*L. monocytogenes* 39-2).

#### 2.4.5. Trials #4 and #5: Surface Application of Filter vs. Pasteurized Bac+ CFS and Neutralized vs. Non-Neutralized CFS in *L. monocytogenes* Challenge Studies on Hotdogs

Several additional modifications of the above were also examined, including a comparison of filter-sterilized vs. pasteurized Bac^+^ CFS preparations and pH-neutralized vs. non-neutralized CFS preparations. A summary of the various trials and treatments received are presented in Table 2.

### 2.5. Statistical Analysis

Shelf life assays were performed in triplicate and means were plotted versus time. The statistics functions in SigmaPlot 13 (Systat Software, San Jose, CA, USA) were used to perform one-way repeated measures analysis of variance (RM-ANOVA) to determine if significant difference exists between different treatments with level of significance set at 0.05 (*p*-value).

## 3. Results and Discussion

### 3.1. Trial #1: Application of Mixed MOA Bac^+^ LAB vs. L. monocytogenes on Hotdogs

**Figure 2:**
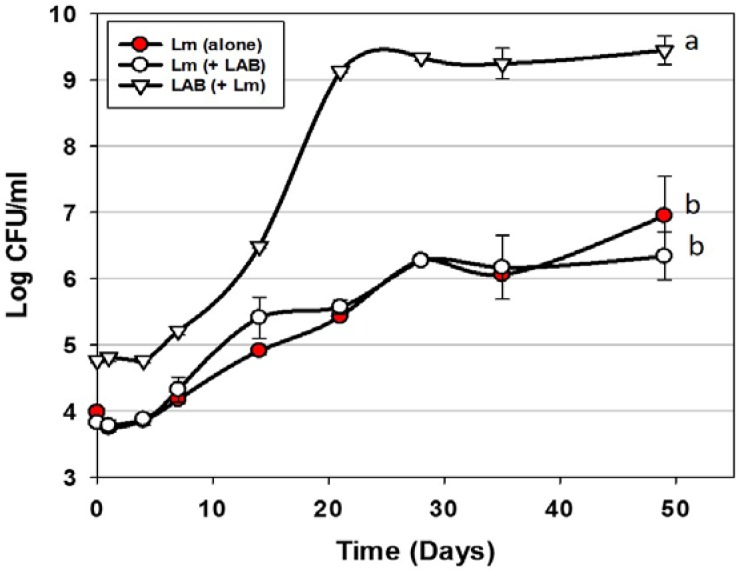
*L. monocytogenes* 39-2 challenge study (hotdogs) with multiple Bac^+^ LAB cultures held at 5 °C for up to 49 days in vacuum packages (Trial #1). *L. monocytogenes* 39-2 was inoculated alone (Lm alone; 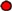) or in combination (Lm+LAB; ○) with 5 Bac^+^ LAB (*En. faecium* FS56-1, *En. thailandicus* FS92, *En. faecium* FS97-2, *En. thailandicus* RP-1, and *Pe. acidilactici* Bac 3). All trials were performed in triplicate replication; data points represent the means and error bars represent standard deviation from the mean. Treatments with different letters are significantly different (repeated measures, *p* < 0.05); those with the same letters are not significantly different (*p* > 0.05).

Trials were performed examining the use of Bac^+^ LAB cultures as protective co-inoculants that comprised the 3 MOAs described previously [20,22]. The Bac^+^ LAB were intentionally added at approximately 1-log higher level than the co-inoculated *L. monocytogenes* 39-2. In preliminary co-inoculation challenge studies with individual Bac^+^ strains, inhibition of *L. monocytogenes* 39-2 was not observed (data not shown). We hoped to demonstrate microbial control of *L. monocytogenes* by mixing cultures comprising the 3 MOAs simultaneously vs. *L. monocytogenes* 39-2. However, we again did not observe any inhibition of *L. monocytogenes* in spite of the additional growth during storage of one or more of the Bac^+^ strains exceeding that of *L. monocytogenes* by >3 logs (Figure 2). It is likely that the storage conditions were unsuitable for the cultures to produce any, or enough, bacteriocin to be inhibitory to *L. monocytogenes* 39-2. Although others have shown control of *L. monocytogenes* on RTE meats using LAB cultures [27,28], we did not observe this effect and demonstrates the difficulty in relying on the use of live competitive cultures to provide inhibitory protection to food products against potential pathogens. Another potential issue with the use of live protective cultures is that the level required for control, or their potential growth during storage, could be the equivalent of spoilage. It should be noted that lactic acid produced during culture growth may be buffered by the food matrix and that bacteriocins are secondary byproducts and their production is not necessarily concomitant with growth.

### 3.2. Trial #2: Listeria Monocytogenes Challenge Studies Using Hotdogs Made with Bacteriocin Extracts Added during Manufacture or Sprayed Post-Cook onto Encased Products

Additional challenge studies were performed using CFS preparations, either added to the meat emulsion during manufacture or by manual spray onto the encased hotdogs after cooking, but before peeling (Figure 1). In prior testing of the heat stability of our bacteriocins, we found that they were able to tolerate high levels of heating, allowing us to use pasteurization to further insure that extracts were free of producer cells. Moreover, bacteriocins would provide a greater potential for application if their thermal tolerance allowed their inclusion in products that may be heated or cooked. 

**Figure 3:**
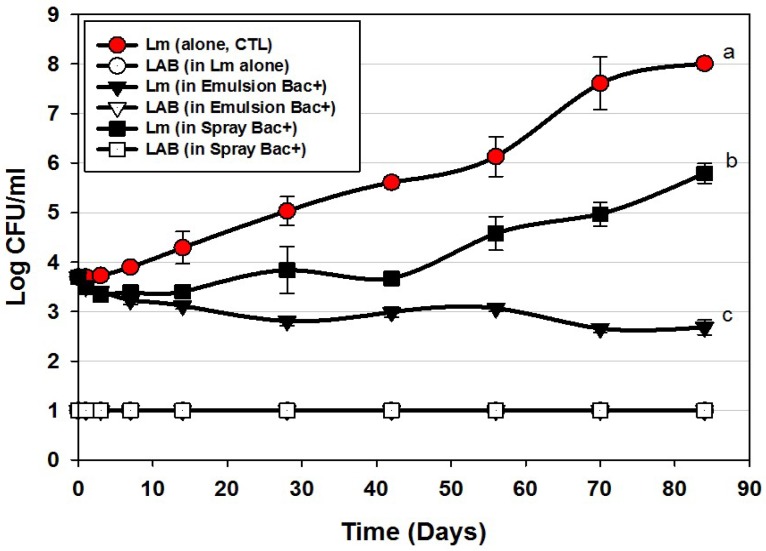
*L. monocytogenes* 39-2 challenge study on hotdogs with multiple MOA Bac^+^ CFS held at 5 °C for up to 84 days in vacuum packages (Trial #2). *L. monocytogenes* 39-2 was inoculated onto untreated hotdogs (Lm, alone), inoculated onto hotdogs in which the bacteriocin mixture was mixed into the meat emulsion during manufacture (in emulsion Bac+), or inoculated onto hotdogs in which the bacteriocin mixture was previously sprayed while hotdogs were still in casings before peeling (in spray Bac+). The Bac^+^ CFS was comprised of curvaticin FS47, curvaticin Beef3, pediocin Bac3, and lacticin FLS1. Platings for LAB from all treatments were made on acidified MRS (hollow symbols). All sample treatments were performed in triplicate replication; data points represent the means and error bars represent the standard deviation from the means. Treatments with different letters are significantly different (repeated measures, *p* < 0.05).

Application of bacteriocins during the manufacture of hotdogs provided excellent control of *L. monocytogenes* 39-2 during the 12 weeks of challenge showing a slight decline almost immediately that continued slowly through 84 days ending at approximately 1-log lower than was initially added (Figure 3). This level of control is exceptional compared to the >4-log increase observed for the control treatment and resulted in a difference of 5-logs. As with most applications that depend on ingredients added to the entire mass of product volume, this treatment required the highest amount of bacteriocin extract added as an ingredient (i.e., bacteriocin extract was approximately 27% (*w*/*w*) of the total emulsion composition). Another consideration that may occur in such applications is that the active bacteriocin may be reduced due to interaction with food matrix components during cooking, as it is known that bacteriocins have hydrophobic motifs that can partition into the fat phase of certain foods [29,30].

In contrast, the cooked and encased product that was sprayed with bacteriocin before peeling showed moderate inhibition relative to the control treatment (Figure 3). Although some bacteriocin probably penetrated the permeable casing, most of it likely washed off. Although a smaller amount was used to spray the encased hotdogs than when included in the meat matrix, one could argue it may be more effective to spray hotdogs after peeling than before peeling, and is reflected in our next approach. In the Bac^+^ CFS challenge with *L. monocytogenes*, we also plated the liquid recovered from packages for potential indigenous lactic acid bacteria. We were careful to pasteurize our cooked, frozen, and thawed hotdogs prior to use in challenge studies and did not want production of lactic acid from potential indigenous bacteria to influence interpretation of inhibition from added bacteriocin. The data indicates that indigenous LAB were below our limit of detection and did not contribute to the inhibition observed. In real commercial applications, of course any further contributory inhibition by lactic acid contributed by indigenous LAB would be welcome to help inhibit potential pathogens such as Listeria. The bacteriocin mixture might have also been inhibitory to potential contaminating LAB as well (i.e., sensitive).

### 3.3. Trials #3, #4, and #5: Listeria monocytogenes Challenge Studies with Multiple-MOA Bacteriocin Extracts Added after Peeling (During Packaging)

Several trials were conducted by adding CFS preparations directly to packages prior to vacuum packaging. These applications utilize the least amount of bacteriocin because they are applied on the product surface after cooking and vacuum packaging can provide a tight space for them to perform as a thin film between food product and packaging film where most surface microorganisms may be found. Most post-process contamination of RTE meats usually occurs on the product surface, as *L. monocytogenes* is mostly a surface problem resulting from contact with contaminated food contact surfaces.

During the progression of our studies, we used a variety of strains that were grouped according to MOA as described earlier. We confirmed the identity of our organisms using 16S rRNA PCR amplification followed by sequencing for both old stock cultures and newly identified strains as reported elsewhere [26]. The use of 16S rRNA analysis had shown that some strains previously identified as *Lactococcus* by API metabolic assays were actually *Enterococcus* [25,26]. *Enterococcus* sp. are commonly isolated from foods [31], some are even used as starter cultures [32] and still others as probiotics [33]. However, their use in foods has been challenged because of their involvement as opportunistic human pathogens [34]. We don’t feel the use of *Enterococcus* strains are a problem with our work because of the use of cell-free extracts rather than live strains. However, we were still interested in seeing if we could develop a repertoire of strains comprised solely of bacteriocin extracts from what is generally considered traditional ‘food-grade’ lactic acid bacteria should cell-free extracts from enterococcal strains become a debilitating issue.

**Figure 4:**
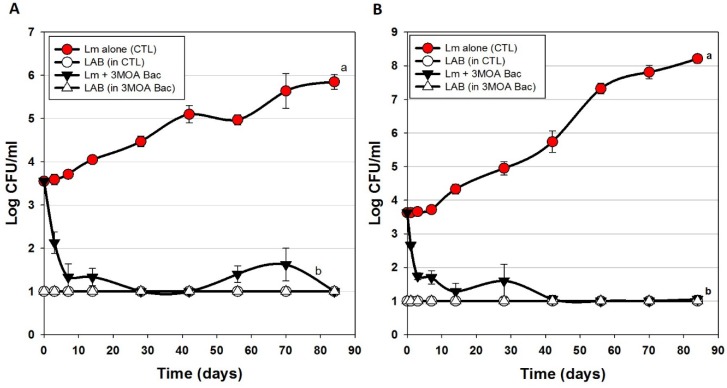
Hotdog challenge study with surface-applied bacteriocin extracts comprising 3 mixed MOAs. *L. monocytogenes* 39-2 was either inoculated onto hotdogs alone (as control) or with added bacteriocin extracts (Trial #3). (**A**) bacteriocin extracts included curvaticin FS47, pediocin Bac3, enterocin FS56-1, and enterocin FS92. (**B**) bacteriocin extracts included curvaticin FS47, pediocin Bac3, enterocin FS56-1, and lacticin FLS1. Plate counts for LAB from these treatments were made on acidified MRS (hollow symbols). All sample treatments were performed in triplicate replication; data points represent the means and error bars represent the standard deviation from the means. Treatments with different letters are significantly different (repeated measures, *p* < 0.05).

Figure 4 represents several hotdog challenge studies whereby we used 2 enterococcal bacteriocins (Figure 4A) and then traded out one of them out for a lactococcal bacteriocin and used only 1 enterococcal bacteriocin (Figure 4B). There was moderate inhibition and control of *L. monocytogenes* when CFS was added to the meat matrix before cooking (Figure 3). However, when Bac^+^ CFS was surface-applied, the data shows a significant drop in *L. monocytogenes* within the first few days of storage (~2 logs) and continues until about 7–10 days showing a stable level of *L. monocytogenes* at or near the limit of detection (Figure 4A,B).

The USDA-FSIS regulations for ‘control of *L. monocytogenes* in RTE meats’ specifies conditions for several risk categories of RTE meats whereby an Alternative 1 process (least risk) is described as possessing a post-process lethality step for *L. monocytogenes* (i.e., ≥1-log reduction) and control of *L. monocytogenes* (i.e., ≤2-log increase) during shelf life [35]. This is often obtained by two separate mechanisms but can also be achieved by a single treatment. The data presented herein may satisfy those requirements for both post-process lethality and control of *L. monocytogenes* during shelf life using a single surface treatment with these bacteriocin preparations.

**Figure 5:**
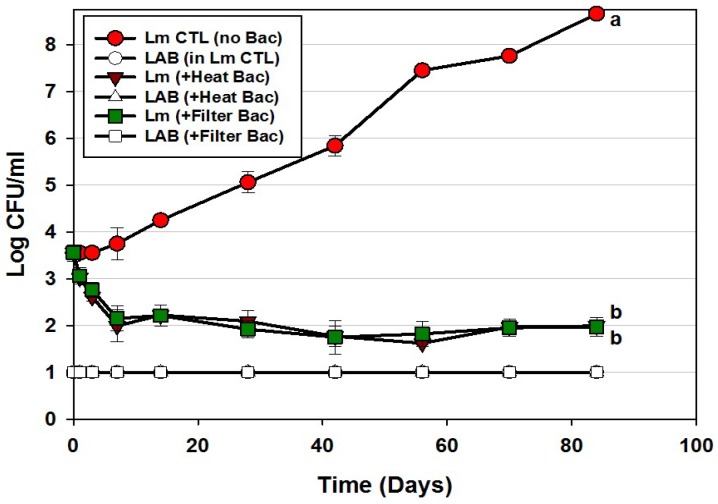
Hotdog challenge study with surface-applied bacteriocin extracts comprising 3 MOAs (curvaticin FS47 and Beef3, pediocin Bac3, and lacticin FLS1; Trial #4). *L. monocytogenes* 39-2 was either inoculated on hotdogs alone, or with added heat-treated or filter-sterilized CFS preparations, vacuum-packaged, and held for up to 12 weeks at 5 °C. Platings for LAB from these 3 treatments were also made on acidified MRS (hollow symbols). All sample treatments were performed in triplicate; data points represent the means and error bars represent the standard deviation from the means. Treatments with different letters are significantly different (repeated measures, *p* < 0.05); those sharing the same letter are not significantly different, *p* > 0.05).

We further examined the use of Bac^+^ CFS preparations as hotdog surface treatments using only traditional lactic acid bacteria from our collection that complied with the 3 mode of actions defined earlier. Using this approach, we also compared the efficacy of filter-sterilized vs. heat-pasteurized bacteriocins (Figure 5). The data appears no different when using filter-sterilized vs. heat-pasteurized bacteriocin extracts demonstrating that heat pasteurization (after centrifugation) imparts no detrimental effect to the bacteriocins and is an easy method of eradicating residual bacteriocin-producer cells (Figure 5). The initial decrease of *L. monocytogenes* was not as dramatic as that observed with the enterococcal bacteriocins (Figure 4). We ascribe this to the moderate production of bacteriocin FLS1 by *Lactococcus lactis* FLS1 compared to that produced by others in the CFS. The enterococcal strains have also been shown to possess genes for multiple enterocins [21]. *L. monocytogenes* increased approximately 5-log in control samples, showing >6.5-log difference between control and treatments. Again, LAB were not detected within the trials and demonstrates that inhibitory action was again solely provided by the added bacteriocins.

**Figure 6:**
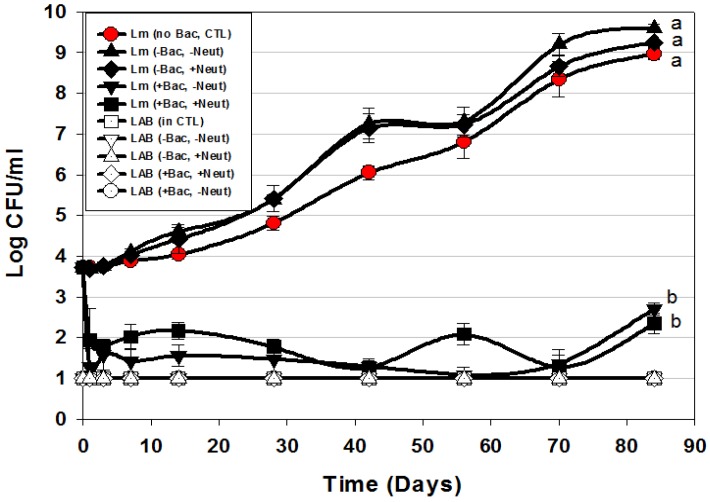
Hotdog challenge study vs. *L. monocytogenes* 39-2 with surface-applied bacteriocin extracts (+Bac) comprising mixed MOAs (curvaticin FS47 and Beef3, pediocin Bac3, and lacticin FLS1) and neutralized (+Neut) vs. non-neutalized (-Neut) culture extracts (Trial #5). *Lb. delbueckii* 4797 was used for bacteriocin-negative (-Bac) CFS extracts that were also used both neutralized (+Neut) and non-neutralized (-Neut). All sample treatments were performed in triplicate replication; data points represent the mean and error bars represent the standard deviation from the mean. Treatments with different letters are significantly different (repeated measures, *p* < 0.05); those sharing the same letter are not significantly different, *p* > 0.05).

Since LAB bacteriocin culture extracts may also contain lactic acid, we further examined the use of neutralized vs. non-neutralized CFS from both Bac^+^ (bacteriocin treatment) and bacteriocin-negative (Bac^−^, control) LAB in order to more confidently assert the inhibition to bacteriocin-related antimicrobial activity (Figure 6). The use of Bac^−^ LAB culture extract (*Lb. delbrueckii* 4797) was an additional control treatment to evaluate whether lactic acid produced by cultures (without the influence of bacteriocin) was contributory to the inhibition observed in these assays as we have observed a contributory effect of lactic acid in culture extracts in microplate *in vitro* assays [22].

The data shows that addition of neutralized (-Bac, +Neut) or non-neutralized (-Bac, -Neut) CFS extracts from a Bac^−^ LAB culture did not show any inhibition of *L. monocytogenes* in comparison to the control treatment in which sterile water was added instead of Bac^−^ culture extracts (Figure 6). Although the treatments whereby CFS from a Bac^−^ culture were added showed no significant difference to *L. monocytogenes* 39-2 to which water was added instead of CFS, they did have slightly higher growth levels, perhaps attributed from additional nutrients from the CFS. The main point was that lactic acid in the Bac^−^ extracts did not inhibit *L. monocytogenes* (Figure 6). In another study using microplate *in vitro* assays, we observed definite lactic acid effects when comparing the effects of neutralized vs. non-neutralized extracts [22]. We suggest that the difference lies in the microplate assay format whereby culture extracts comprised approximately 30% of the assay volume in prior *in vitro* assays [22] whereby in these assays the added culture extracts comprised <0.1% of the package weight which contained substantial organic material (i.e., hotdogs) that can readily buffer the effects of organic acids from even non-neutralized extracts. Addition of the multi-MOA bacteriocin mixture resulted in approximately a 2-log reduction of *L. monocytogenes* within the first 1–3 days of addition that did not show any increase (beyond the initial added level) during the 12-week challenge study, showing ~7-log difference from controls. These data again suggest that these treatments satisfy the requirements for USDA-FSIS Alternative 1 product classification for RTE meat and poultry products [35].

## 4. Conclusions

The data presented herein shows the efficacious application of a mixture of bacteriocins against *L. monocytogenes*. The bacteriocin-producing strains were isolated from various sources including foods found in supermarkets [25,26], however it was the unique method of procuring and using spontaneous resistant mutants as ‘microbial screens’ to categorize them into different modes of action (MOA) [20,21,22] and then using a mixture comprising those different MOAs in order to provide an effective cocktail of natural antimicrobial bacteriocins that active synergistically to inhibit *L. monocytogenes* in RTE meats as observed in this study. Although others are also using surface application of bacteriocins [36], we feel that the multiple MOA approach works well to minimize the possible development of spontaneous bacteriocin-resistant mutants that readily occurs with bacteriocins of similar MOA [20,21]. Since our bacteriocins are heat resistant, they can be added in, or on, foods that may be heated or cooked. The use of culture extracts provides an opportunity for the biological activity to be standardized whereas the use of live cultures to provide antimicrobial protection in non-actively growing situations is tenuous. We feel that such bacteriocin extracts produced by food-grade LAB may be used freely as food ingredients to act as food preservatives (i.e., biopreservatives) in RTE meats and other products where they show proven efficacy against targeted pathogens and susceptible spoilage organisms.

## Figures and Tables

**Table 1:** Bacterial strains used in this study.

Microorganism	Strain Designation	Source/Reference
*Lactobacillus delbrueckii*	4797-2	Muriana culture collection
*Listeria monocytogenes*	39-2 (R0)	[20,21,22,23]
*Lactobacillus curvatus*	FS47	[25]
*Lactobacillus curvatus*	Beef 3	[26]
*Pediococcus acidilactici*	Bac 3	[26]
*Enterococcus faecium*	FS56-1	[21,25,26]
*Lactococcus lactis*	FLS-1	[26]
*Enterococcus thailandicus*	RP-1	[21,26]
*Enterococcus thailandicus*	FS92	[21,25]

**Table 2:** Description of trials and treatments used in this study applying bacteriocin-producing cultures (Bac^+^) or their cell free supernatants (CFS) to inhibit *L. monocytogenes* on RTE meats.

Trial	Description of Treatment	Data
Trial 1	Use of bacteriocin-producing (Bac^+^) cultures vs. *L. monocytogenes*	Figure 2
Trial 2	Bac^+^ CFS added into meat matrix before cooking	Figure 3
	Bac+ CFS sprayed onto hotdogs in casings before peeling	
Trial 3	Bac^+^ CFS as surface treatment (includes CFS from 2 *Enterococcus* strains)	Figure 4A
	Bac^+^ CFS as surface treatment (includes CFS from 1 *Enterococcus* strain)	Figure 4B
Trial 4	Bac^+^ CFS as surface treatment: All CFS was from traditional lactic acid bacteria; filter vs. heat-pasteurized Bac^+^ CFS	Figure 5
Trial 5	Bac^+^ CFS as surface treatment: Neutralized vs. non-neutralized CFS and Bac^+^ vs. Bac^−^ CFS	Figure 6
